# Evaluation of sepsis induced cardiac dysfunction as a predictor of mortality

**DOI:** 10.1186/s12947-018-0149-4

**Published:** 2018-11-30

**Authors:** Amarja Ashok Havaldar

**Affiliations:** MICU, St John’s Medical College, Kormangala, Bangalore, 560034 India

**Keywords:** Sepsis, Septic cardiomyopathy, Troponin I, Vasoplegia

## Abstract

**Background:**

Sepsis is characterized by life threatening organ dysfunction with dysregulated immune response. Cardiac dysfunction seen in sepsis is unique as it is reversible within 7–10 days. Initial study by Parker et al. in 1984, showed, paradoxically lower ejection fraction in survivors of septic shock. Subsequent meta-analysis did not support that survivors had lower ejection fraction. Aim of our study was to assess the sepsis induced cardiac dysfunction by 2D echocardiography and Troponin I.

**Methods:**

After obtaining institutional ethical committee approval (ref 125/2016), a prospective observational study was done in an university medical college from February 2016 to April 2016. Inclusion criteria were patients diagnosed with sepsis by new sepsis definition. Pregnant patients and patients with poor echo window were excluded. Echocardiographic assessment was done within 48 h of diagnosis of sepsis by standard methods. Primary outcome was ICU mortality and secondary outcome was ICU length of stay. Statistical analysis was done using STATA™ (Version14, College station TX).

**Results:**

Fifty eight patients were screened, ten were excluded due to poor echo window. Baseline characteristics were similar in survivors and non survivors, except APACHE II, SOFA age and cumulative fluid balance. Echocardiographic parameters, mitral annular plane systolic excursion (MAPSE), E/e’ and LV systolic function assessed by visual gestalt method were found to be statistically significant. Parameters found significant in bivariate analysis were used as a covariate in logistic regression. APACHE II and MAPSE were significant co-variates in logistic regression with ROC (0.95) and calibration was satisfactory (chi2(df8),1.98, *p* = 0.98).

**Conclusions:**

Sepsis induced cardiac dysfunction assessed by echocardiography showed measurement of MAPSE when combined with APACHE II was a good predictor of mortality. Among the echocardiographic parameters MAPSE alone was a good predictor of mortality. Results of this study need further validation from larger study.

## Background

Sepsis is characterized by dysregulated immune response with life threatening organ dysfunction [1–3]. This is one of the major cause of mortality in intensive care unit. Septic shock is characterized by low systemic vascular resistance and it has two phases of shock. Early hyperdynamic phase of septic shock has high cardiac output and late hypodynamic phase is characterized by reduced cardiac output [4–8]. Cardiac dysfunction seen in patients with sepsis can be of different types, systolic, diastolic or both and etiology of sepsis may affect the type of cardiac dysfunction. To explain it further, sepsis due to underlying lung pathology can result in elevation of right ventricular (RV) afterload. High RV afterload leads to less pulmonary blood flow and reduces the possibility of left ventricular (LV) diastolic failure if LV function is normal prior to sepsis [4, 9–12].

Sepsis induced cardiac dysfunction was first described by Parker et al., in the study of 20 patients. These patients had hematological and solid organ malignancies and survivors had lower ejection fraction which was considered as an adaptive response to overcome vasoplegia [4–6].

However, recent meta-analysis on sepsis induced ventricular dysfunction showed no difference in ejection fraction (EF) between survivors and non survivors [13].

Cardiac specific biomarker such as Troponin I has been studied in septic patients and elevated Troponin I was associated with higher risk of mortality [14, 15]. The aim of the current study was to assess prognostic significance of sepsis induced cardiac dysfunction evaluated by echocardiography and Troponin I.

## Methods

Formal consent from institutional ethical committee was obtained prior to initiating the study (ref no125/2016). It was a prospective observational study conducted for a duration of 3 months from February 2016 to April 2016 in the 30 bedded ICU of a large university medical center. Patients were included, after obtaining consent from patients or legally acceptable representatives. All patients above 18 years of age with the diagnosis of sepsis were included. Patients with poor echo window and pregnant patients were excluded from the study. A convenience sampling method was used for patient selection.

Baseline characteristics, APACHE II and SOFA scores were calculated within 24 h of ICU admission. APACHE II score takes into account, clinical, laboratory, age and comorbidities. It indicates severity of illness. Similarly, SOFA score considers number of organ failures. Echocardiographic parameters and Troponin I were collected within 48 h of diagnosis of sepsis. All patients were followed up till the ICU discharge and the primary outcome of interest was ICU mortality.

Echocardiographic measurements were done using a Sonosite™ Edge portable machine with a 2.5 MHz probe and inbuilt cardiac calculation software. LV systolic function was assessed by visual gestalt method, Simpson’s method, fractional shortening and MAPSE. By visual gestalt method, cardiac function was classified into hyperdynamic, normal, mild, moderate, and severe LV dysfunction. By Simpson’s method readings were taken in apical 4 chamber view and EF was calculated. LV fractional shortening was assessed by M-mode echocardiography. By using M- mode at lateral mitral annulus MAPSE was measured. LV diastolic dysfunction was assessed by transmitral flow velocity (E, A and deceleration time DCT) were measured. E/A was calculated. Assessment of mitral annular flow velocity was done using tissue doppler imaging. Lateral mitral annular flow velocities, e’ and a’ were measured. E/A, E/e’ were calculated. In patients having regional wall motion abnormalities (RWMA), both lateral and septal mitral leaflet annular velocities were measured and averaged. The right ventricular systolic function was assessed using tricuspid annular plane systolic excursion (TAPSE). Tei index was calculated which is a measure of myocardial performance. Ventriculoarterial elastance was calculated by taking the ratio of end systolic volume (ESV) by stroke volume (SV). All measurements were performed by the first author to avoid inter-observer bias. An average of consecutive three recordings was taken to avoid intra-observer bias.

Statistical analysis was done using STATA™ (Version14, College station TX). Data was expressed as mean (SD) or median (Interquartile range IQR) as appropriate. Continuous variables were analysed by ‘t’ test (Satterwhaite’s for unequal variance) and categorical variables were analysed using ‘χ^2^’ test. ‘Mann-Whitney U’ test was used for non-parametric continuous variables. A standard 95% confidence limit and a *p* value at 0.05 were used for assessing statistical significance. Variables identified as significant in the bivariate analysis were used as independent variables and outcome was used as dependent variable to develop a predictive model using logistic regression.

## Results

Fifty eight patients were screened. Ten patients (17.24%) were excluded due to poor echo window. Out of fourty eight patients, 29 patients (60.41%) survived and 19 patients (39.58%) died. Among the baseline characteristics, age (65.94 vs 54.89), APACHE II (25.84 vs 16.34), SOFA (9 vs 4) and higher cumulative fluid balance of first 3 days were statistically significant. Preexisting comorbidities did not had any effect on the outcome (Table 1).

**Table 1:** Baseline characteristics

Baseline Parameters	Survivors *n* = 29	Non Survivors *n* = 19	*P* value
Age	54.89(12.61)	65.94(11.51)	0.003^*^
APACHE II	16.34(4.91)	25.84(6.44)	< 0.001^*^
SOFA	4(3-6)	9(8–12)	< 0.001^*^
Fluid balance	975(− 182–2495)	3654(3026.7–5848)	< 0.001^*^
Lactate	2.10(1.2-4.4)	6.3(2.10–7.20)	0.06
Troponin Day 1	0.03(0.01–0.11)	0.08(0.04–1.68)	0.0503
Comorbidities			
Diabetes Mellitus	10(34.48)	9(47.36)	0.37
IHD	4(13.33)	5(27.77)	0.27
Hypertension	9(31.03)	8(42.10)	0.43
CKD	2(6.89)	2(10.52)	0.65
CLD	3(10.34)	0(0)	0.14
COPD	1(3.44)	0(0)	0.41

^*^significant *p* < 0.05

Cardiac biomarker Troponin I was not statistically significant (0.08 vs 0.03) in both groups (*p* = 0.051) (Table 1). Echocardiographic parameters were similar among survivors and non survivors except for the MAPSE, E/e’ and LV systolic dysfunction assessed by visual gestalt method. Assessment of LV systolic function by this method showed hyperdynamic LV (12.2%), normal (26.83%), mild (19.51%), moderate (26.83%) and severe LV dysfunction in (14.63%) patients (Table 2). Severe LV systolic dysfunction was seen in nonsurvivors as compared to survivors.

**Table 2:** Echocardiographic parameters

ECHO parameters	Survivors (29)	NonSurvivors (19)	*p* value
EF M mode	58.73(9.85)	59.08(19.48)	0.52
EF Simpson’s Method	30.85(14.26)	36.31(13.98)	0.89
MAPSE	1.54(1.39–1.69)	1.12(0.90–1.35)	0.026^*^
LV systolic function Visual Gestalt			0.02^*^ Fischer’s Exact
Hyperdynamic	3/5	2/5	
Normal	10/11	1/11	
Mild	7/8	1/8	
Moderate	7/11	4/11	
Severe	1/6	5/6	
E/A	1.04(0.40)	1.30(0.59)	0.95
DCT(ms)	122.63(43.35)	128.22(55.30)	0.71
S (cm/sec)	9.83(2.98)	10(3.58)	0.87
e’ (cm/sec)	11.22(2.70)	10.27(3.21)	0.30
E/e’	6.32(1.75)	8.03(2.79)	0.025^*^
ESV (ml)	47.1(36–79.8)	45.95 (27.45–73.80)	0.72
SV(ml)	27(14–37.1)	23.9(13.7–32.15)	0.65
EDV(ml)	69(58.4–104.6)	70.65(56.2–115.70)	0.83
VTI (cm/sec)	15.14(3.13)	13.93(3.19)	0.19
VA elastance	2.42(1.46–3.78)	1.68(1.38–3.18)	0.67
Tei Index	0.61(0.19)	0.64(0.27)	0.635

^*^significant *p* < 0.05

Multivariable logistic regression analysis was done using echocardiographic parameters which were found statistically significant in bivariate analysis. It showed MAPSE was the only significant parameter (Table 3).

**Table 3:** Multivariable logistic regression of echo parameters

Mortality	Coefficient (β)	SE	z	Odds Ratio	*p* value	CI
MAPSE	−3.7	1.71	−2.17	0.024	0.030	0.00–0.69
E/e’	0.31	0.25	1.23	1.37	0.219	0.82–2.29
LV systolic Visual Gestalt	0.44	0.40	1.09	1.56	0.275	0.70–3.47
Constant	1.21	2.8		3.37	6.72	

^*^significant *p* < 0.05

**Fig. 1:**
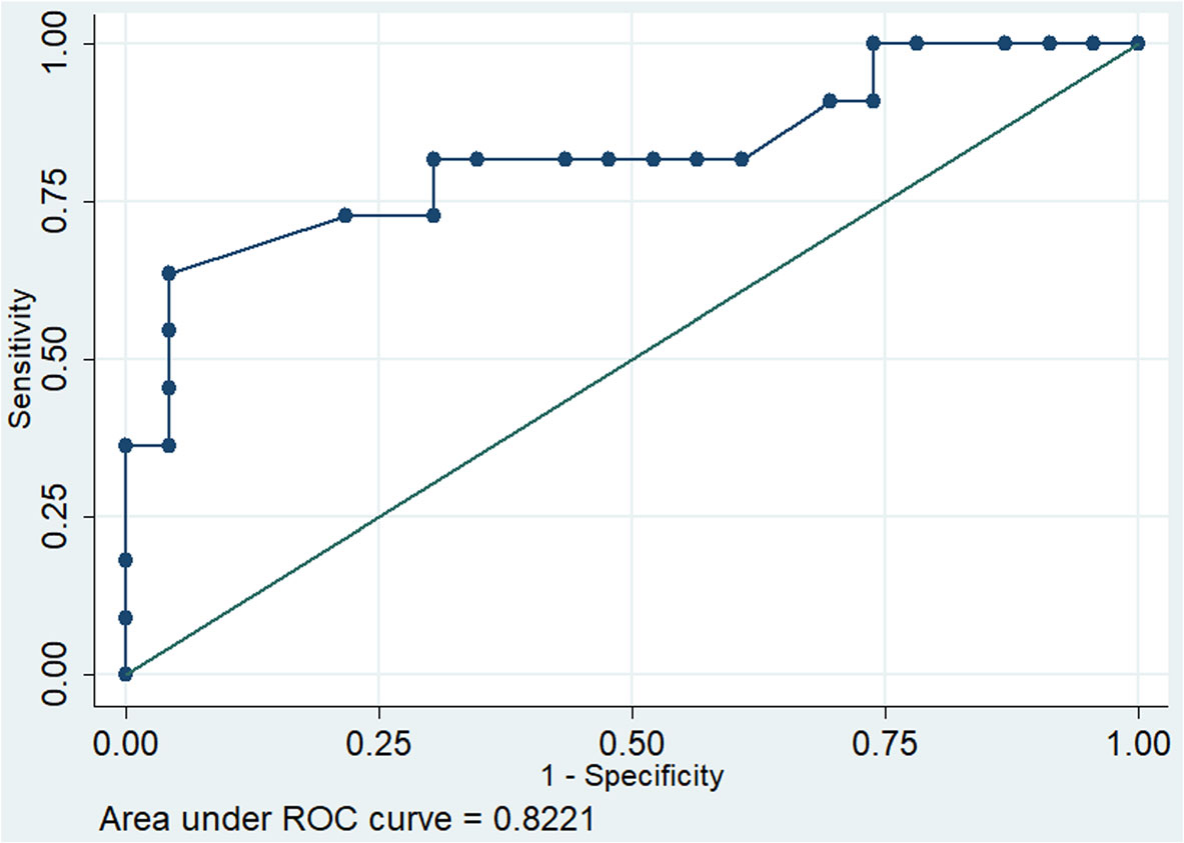
Receiver operating characteristics of MAPSE

**Fig. 2:**
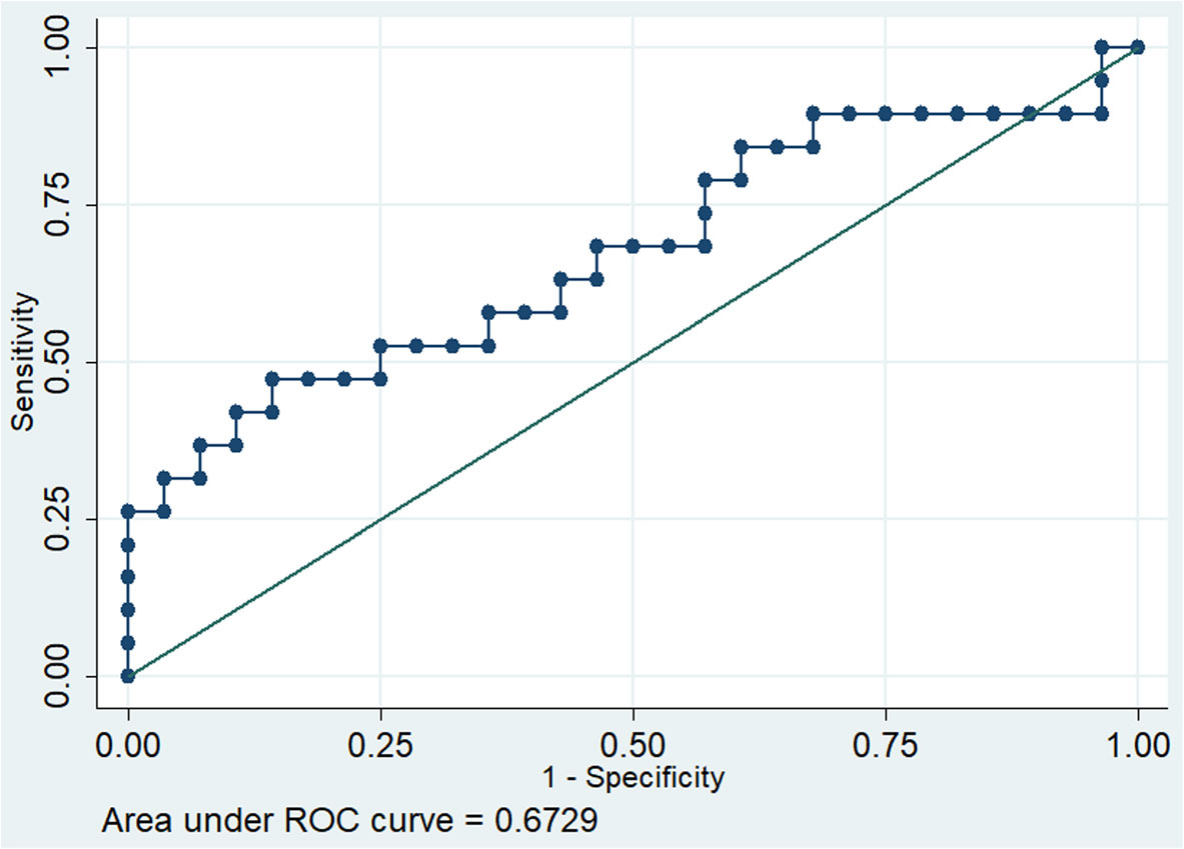
Receiver operating characteristics of E/e`

**Fig. 3:**
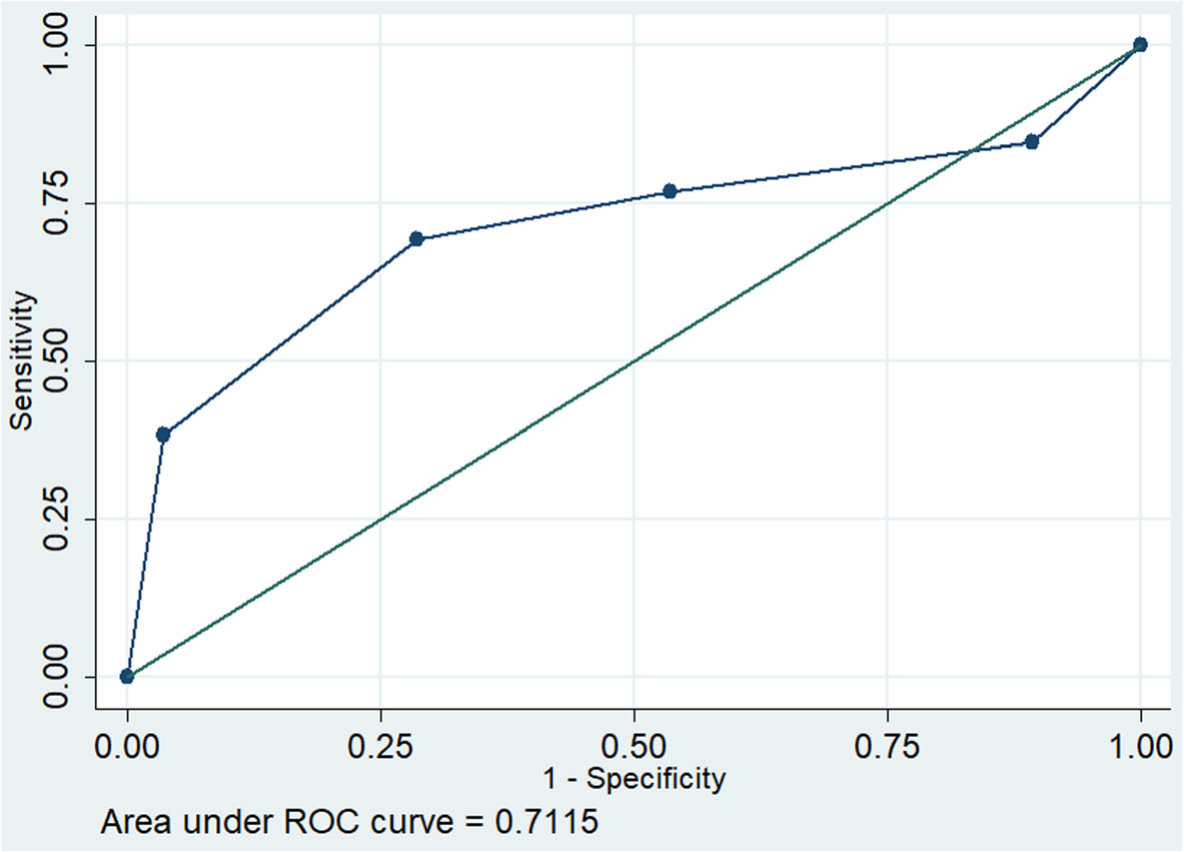
Receiver operating characteristics of LV systolic function

The receiver operating characteristics (ROC) for parameter MAPSE showed good discrimination (Area under the curve [AUC 0.822]) as against poor discrimination for E/e’ (AUC 0.67) and fair discrimination for LV systolic function (AUC 0.71) (Figs. 1, 2 and 3).

**Fig. 4:**
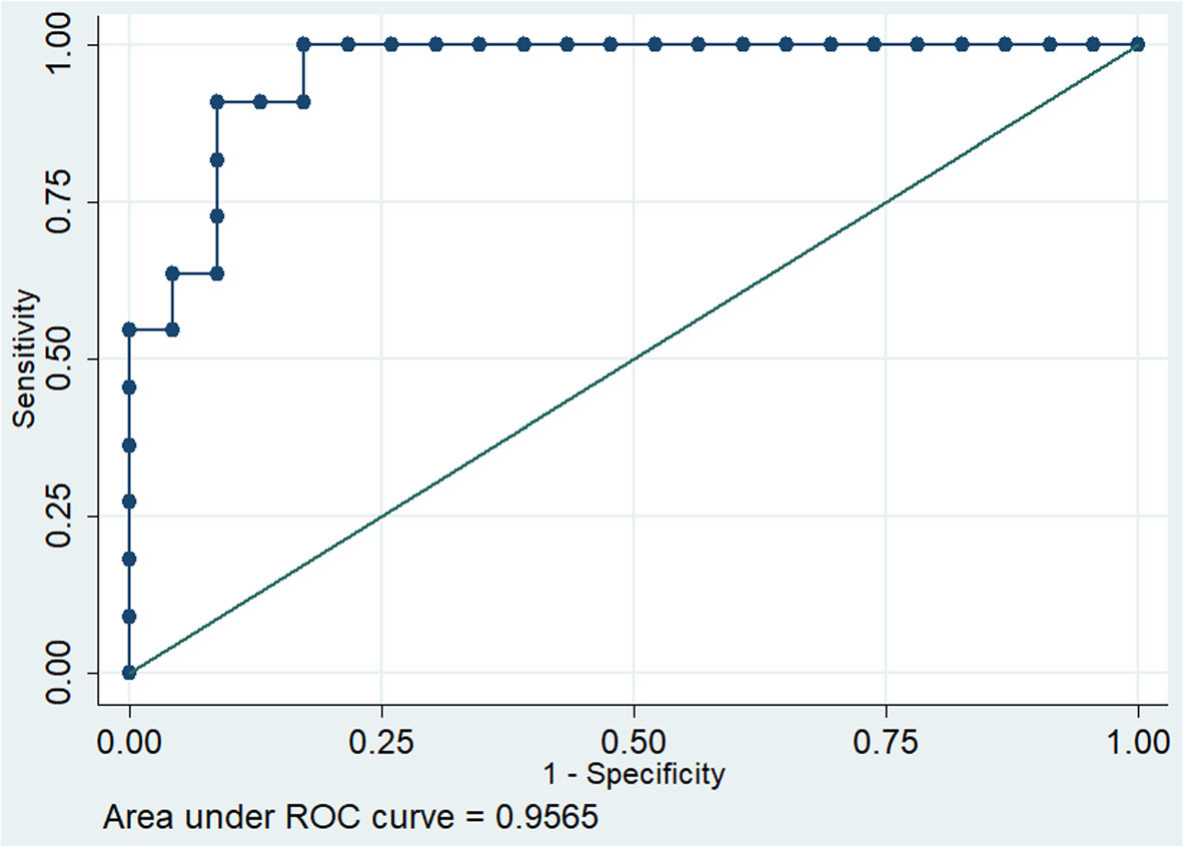
Receiver operating characteristics of MAPSE and APACHE II

**Fig. 5:**
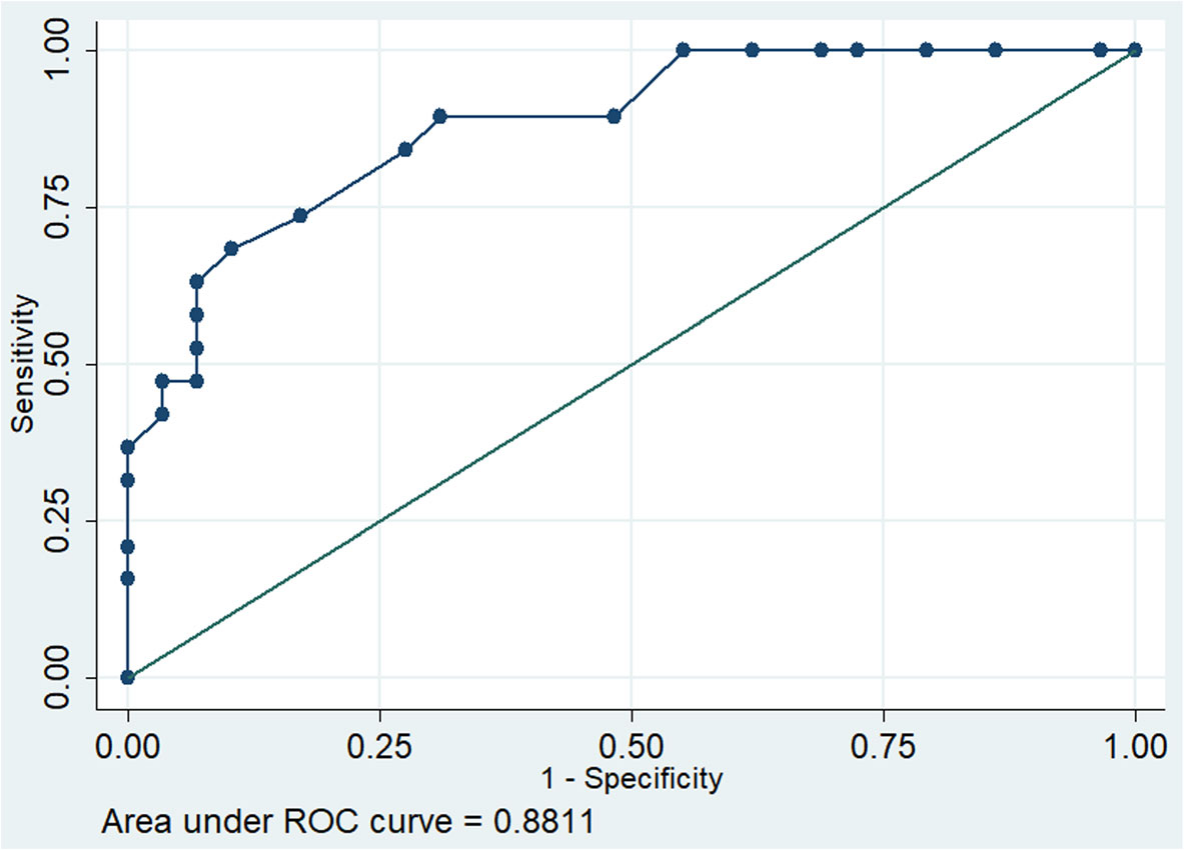
Receiver operating characteristics of APACHE II

In developing a predictive model using echocardiographic and clinically important parameters we found only APACHE II and MAPSE were found to be significant co-variates. (APACHE II coef 0.4869 *p* = 0.02, MAPSE -9.3672 *p* = 0.04, constant 0.7109). The discrimination of this model was excellent (receiver operating characteristics [ROC 0.95]) and calibration was satisfactory (chi2(df8),1.98, *p* = 0.98) (Fig. 4) as against each parameter when taken individually. (ROC for APACHE was AUC 0.88 Fig. 5).

Among the echocardiographic parameters, MAPSE alone was found to be the predictor of mortality with coef − 3.7, *p* = 0.03 and constant 1.21.

## Discussion

Sepsis induced cardiac dysfunction was evaluated in fourty eight patients. Echocardiographic parameters such as MAPSE, E/e’ and LV systolic dysfunction by visual gestalt were the significant parameters. In multivariate analysis MAPSE and APACHE II were significant covariates. In our study MAPSE when combined with APACHE II could predict the outcome in patients of sepsis.

Initial small study of 20 patients evaluating sepsis induced cardiac dysfunction showed patients with lower ejection fraction had better survival. Patients included in the study were predominantly elderly and with hematological or solid organ malignancy. Repeat echocardiographic assessment after day 7 showed improved ejection fraction in survivors, thereby suggesting reversibility of sepsis induced cardiac dysfunction [6]. Subsequent meta-analysis done showed no difference in ejection fraction among survivors and non survivors. It also showed non indexed left ventricular parameters were mildly increased in survivors. The indexed parameters were obtained by dividing the echocardiographic parameter by body surface area (BSA). The results of the metaanalysis were not easily generalizable due to wide heterogeneity [13].

In earlier studies cardiac function was assessed by invasive methods [6]. Echocardiography has helped to evaluate cardiac function noninvasively and at repeated intervals during each stage of disease process to decide timely intervention [16–19]. In our study, echocardiographic assessment of LV systolic function was done by different methods, which showed MAPSE as the significant covariate in logistic regression. MAPSE is easy to measure and interpret and is indicative of assessment of longitudinal systolic function. In patients with impaired systolic function had lower values of MAPSE [20–22]. Previous studies have found good correlation between MAPSE and left ventricular longitudinal strain [20–22].

In prospective cohort of 50 patients, MAPSE and SOFA score was found to be a good predictive model [20]. Similar findings were seen in the current study, and MAPSE along with APACHE II score was a good predictive model in patients with sepsis.

Cardiac biomarker such as Troponin I elevation in sepsis is due to inflammatory and endothelial factors. It is also influenced by vasopressor support and concomitant renal injury [13]. Elevation of cardiac biomarker, Troponin I in sepsis patients had higher risk of death (OR 1.92) as shown by the meta-analysis [14]. In our study Troponin I was not found to be a significant parameter.

There were certain limitations to our study. This was single centre study. All observations were collected by single observer. To minimize intraobserver variability, average of the three readings were obtained. The assessment of cardiac function was done within 48 h of admission. The patients developed cardiac dysfunction after 48 h were not included in the study. Effect of duration of sepsis and changes in echocardiographic parameters could not be tested. There was only one echocardiographic assessment, so reversibility of cardiac dysfunction could not be evaluated. Echocardiographic assessment was also influenced by use of vasopressors or inotropic agents. Systolic function assessment by MAPSE evaluates mainly longitudinal LV function. It’s role in predicting global LV function needs to be evaluated in further studies. Measurement of continuous cardiac output would have helped in studying the evolution of shock and effect of different therapeutic interventions over a period of time.

## Conclusion

This small study has shown that MAPSE when combined with APACHE II score is a good predictor of mortality. Among the echocardiographic parameters MAPSE alone was a good predictor of mortality. Cardiac biomarker Troponin I was not the significant parameter in our study. Assessment of cardiac function in sepsis patient is important and serial evaluation and measuring of the MAPSE may help to monitor the progress of the patient. Future studies involving more number of patients and multiple observers is indicated to validate results of this study.

## Abbreviations

APACHE: Acute physiology, age and chronic health evaluation
AUC: Area under the curve
DCT: Deceleration time
EF: Ejection fraction
ESV: End systolic volume
ICU: Intensive care unit
IQR: Interquartile range
LV: Left ventricle
MAPSE: Mitral annular plane systolic excursion
ROC: Receiver operating characteristics
RV: Right ventricle
RWMA: Regional wall motion abnormalities
SD: Standard deviation
SOFA: Sequential organ failure assessment
SV: Stroke volume
TAPSE: Tricuspid annular plane systolic excursion
VTI: Velocity time integral
